# Current drivers and geographic patterns of HIV in Lesotho: implications for treatment and prevention in Sub-Saharan Africa

**DOI:** 10.1186/1741-7015-11-224

**Published:** 2013-10-16

**Authors:** Brian J Coburn, Justin T Okano, Sally Blower

**Affiliations:** 1 Center for Biomedical Modeling, Semel Institute of Neuroscience and Human Behavior, David Geffen School of Medicine, University of California, 10940 Wilshire Blvd, Suite 1450, Los Angeles, CA 90024, USA

**Keywords:** HIV, Epidemiology, Risk factors, Circumcision, Geography

## Abstract

**Background:**

The most severe HIV epidemics worldwide occur in Lesotho, Botswana and Swaziland. Here we focus on the Lesotho epidemic, which has received little attention. We determined the within-country heterogeneity in the severity of the epidemic, and identified the risk factors for HIV infection. We also determined whether circumcised men in Lesotho have had a decreased risk of HIV infection in comparison with uncircumcised men. We discuss the implications of our results for expanding treatment (current coverage is only 60%) and reducing transmission.

**Methods:**

We used data from the 2009 Lesotho Demographic and Health Survey, a nationally representative survey of 3,849 women and 3,075 men in 9,391 households. We performed multivariate analysis to identify factors associated with HIV infection in the sexually active population and calculated age-adjusted odds ratios (aORs). We constructed cartographic country-level prevalence maps using geo-referenced data.

**Results:**

HIV is hyperendemic in the general population. The average prevalence is 27% in women and 18% in men, but shows substantial geographic variation. Throughout the country prevalence is higher in urban centers (31% in women; 21% in men) than in rural areas (25% in women; 17% in men), but the vast majority of HIV-infected individuals live in rural areas. Notably, prevalence is extremely high in women (18%) and men (12%) with only one lifetime sex partner. Women with more partners have a greater risk of infection: aOR 2.3 (2 to 4 partners), aOR 4.4 (≥5 partners). A less substantial effect was found for men: aOR 1.4 (3 to 6 partners), aOR 1.8 (≥7 partner). Medical circumcision protected against infection (aOR 0.5), traditional circumcision did not (aOR 0.9). Less than 5% of men in Lesotho have been medically circumcised; approximately 50% have been circumcised using traditional methods.

**Conclusions:**

There is a substantial need for treatment throughout Lesotho, particularly in rural areas where there is the greatest burden of disease. Interventions aimed at reducing the number of sex partners may only have a limited effect on reducing transmission. Substantially increasing levels of medical circumcision could be very effective in reducing transmission, but will be very difficult to achieve given the current high prevalence of traditional circumcision.

## Background

Globally, 33 million individuals are infected with HIV; two-thirds live in Sub-Saharan Africa. The most severe HIV epidemics worldwide occur in Lesotho, Botswana and Swaziland where approximately 25% of the general population are infected with HIV. Notably, this is substantially greater than other Sub-Saharan African countries; for example, in Zimbabwe the prevalence is 14%, in Malawi 11% and in Uganda 7% [1]. Many previous studies have focused on the HIV epidemics in Botswana [2] and Swaziland [3-6]. However, despite the severity of Lesotho’s epidemic, it has received little attention. The most current estimates from Lesotho indicate that 40% of treatment-eligible individuals are not receiving antiretrovirals (ARVs) and that approximately 50% of deaths are attributable to HIV/AIDS [7-9]. In order to optimize the expansion of treatment programs in Lesotho, it is important to determine the within-country heterogeneity in the severity of the HIV epidemic. Here, we have determined the current geographic distribution of the HIV-infected population in Lesotho and constructed gender-specific cartographic maps based on prevalence. We have also identified the risk factors that are driving the epidemic and determined whether male circumcision has reduced the risk of acquiring HIV. We note that clinical trials in other parts of Africa (Kenya, South Africa and Uganda) have shown that medical circumcision protects against HIV infection [10-12]. We discuss the implications of our results for both the expansion of treatment programs and for identifying effective interventions for reducing HIV transmission in Lesotho.

**Figure 1:**
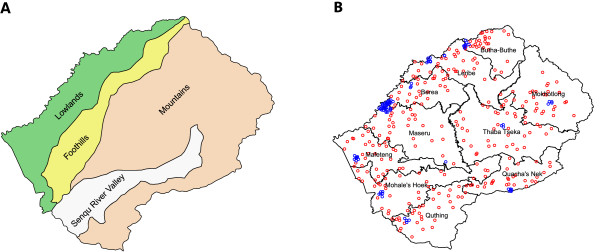
**Maps of Lesotho’s ecological zones and healthcare districts. (A)** Lesotho has four ecological zones: Foothills, Lowlands, Mountains and the Senqu River Valley. **(B)** Map of Lesotho’s 10 healthcare districts with demographic and health survey cluster points for urban (blue) and rural (red) locations.

Lesotho is landlocked within the Republic of South Africa. It is a small (approximately 30,000 km^2^) mountainous country with a population of approximately two million people; altitude ranges from approximately 1,400 m to the highest peak at approximately 3,500 m [13]. The country consists of four ecological zones based on altitude and agriculture: the Lowlands, the Foothills, the Mountains and the Senqu River Valley (Figure 1A). Over half of the population resides in the Lowlands, with the remainder dispersed throughout the other three ecological zones. The mountainous landscape and limited road infrastructure throughout the country make travel difficult and restrict access to healthcare facilities. The country is divided into 10 healthcare districts: Berea, Butha-Buthe, Leribe, Mafeteng, Maseru, Mohale’s Hoek, Mokhotlong, Quacha’s Nek, Quthing and Thaba Tseka (Figure 1B). The capital city (Maseru) has the same name as the healthcare district that contains the city. The Government of Lesotho has called for HIV intervention efforts to be decentralized and implemented at the national, district, community and village levels [8]. We determine the current within-country heterogeneity in the severity of the epidemic at the level of the healthcare district.

In recent years, Lesotho has made significant progress in the rollout of HIV testing and treatment. The Government’s stated aim in their *National HIV and AIDS Strategic Plan (2006–2011)* was to provide rapid expansion of HIV programs. The plan aimed to provide antiretroviral therapy to 80% of those who met the treatment eligibility criteria (that is, with CD4 cell counts less than 350 cells/μL) and to achieve routine testing at all health facilities of more than 80% of individuals above the age of 12 [8]. The plan also detailed comprehensive strategies for providing integrated health services for HIV/AIDS, tuberculosis (TB) and non-HIV sexually transmitted infections (STI). Treatment coverage expanded and testing increased substantially over the specified time period (2006 to 2011); coverage rose from <5% to nearly 60%, and testing increased from 15% to 69% for women and from 11% to 39% for men [9]. However, the national testing and treatment targets of 80% were not reached. Increasing testing rates and expanding treatment coverage remains a major challenge in Lesotho.

## Methods

We used data collected in the Lesotho Demographic and Health Survey (DHS) conducted between 2009 and 2010. This is a nationally representative survey; 9,994 households were sampled and 9,391 of these households participated. The sample was based on Lesotho’s 2006 population census [13,14]. It was designed to allow for separate estimates of demographic and health indicators for: (i) the country as a whole, (ii) urban and rural areas, (iii) the four ecological zones and (iv) the 10 healthcare districts. The survey utilized a two-stage cluster sampling design. In the first stage, 400 clusters (94 urban and 306 rural) were selected from a list of enumeration areas such that the geographic population sample was proportional to the Lesotho 2006 Population and Housing Census [13]. Whether a DHS cluster was identified as urban or rural was defined by the DHS based on Lesotho’s 2006 census that classified enumeration areas and labeled them as urban or rural. Over the past seven years the classification of enumeration areas has not changed; consequently, the urban–rural definition of clusters remains the same. The geographic location of clusters is shown in Figure 1B: blue dots show urban clusters and red dots show rural clusters.

In the second stage of the survey a complete listing of households was created for each cluster. Households were then systematically selected for participation in the survey. A representative from each household completed a questionnaire about all of the household members. This resulted in a dataset consisting of 44,546 individuals; 33,719 were considered to be household members by the DHS based on whether they slept there the night before the survey. Of these individuals, women 15 to 49 years old and men 15 to 59 years old were asked to participate in an individual-level survey. Ninety eight percent of the 7,786 eligible women and 95% of the 3,493 eligible men participated. In addition, HIV tests were conducted on 94% of the eligible women and 88% of the eligible men. For each individual, their test result was linked to their survey data. We used sample weights provided by the DHS so that estimates are representative of Lesotho’s general population. A detailed description of the DHS dataset can be found elsewhere [15].

We estimated age-stratified prevalence (based on five-year age classes) for women 15 to 49 years old and men 15 to 59 years old. We used population estimates from the 2006 Census and calculated prevalence using the DHS data. We also calculated the proportion of the population who were under 15 years old at the time of the survey.

The DHS dataset includes longitude and latitude coordinates for 395 of the 400 cluster locations. Cluster locations contain data from 15 to 25 households and from 41 to 152 individuals. We used indicator data from the household and individual-level surveys to identify the cluster where the data had been collected. Consequently, we were able to geo-reference the survey data and the linked serostatus data. To examine the geography of the epidemic we used the geo-referenced serostatus data and determined HIV prevalence based on the type of residency (urban versus rural), the healthcare district and the ecological zone. For each district, we calculated the proportion of individuals living in the urban center(s) and rural areas. Census data were used to estimate the population size of urban centers. All geographic results were stratified by gender and plotted as cartographic maps.

To identify drivers of the epidemic, we analyzed demographic and behavioral data from the DHS for women 15 to 49 years old and men 15 to 59 years old who were tested for HIV when the data were collected. This resulted in a sample of 3,849 women and 3,075 men. We first conducted a univariate analysis of these individuals to obtain a basic description of HIV in the general population. We did this by calculating HIV prevalence and 95% confidence intervals (CIs) based on gender, age, education, employment status, marital status, religion, sexual activity and pregnancy status. We then conducted a bivariate analysis of the subset of individuals who were sexually active (defined by the DHS as individuals who reported at least one lifetime sex partner) to identify factors associated with HIV infection using a logistic regression model. This resulted in a sample of 3,243 women and 2,546 men. In our regression analysis, we included nonsexual (for example, alcohol use, injections and tobacco use) and sexual (for example, condom use, lifetime number of sex partners, transactional sex and infection with a non-HIV STI in the last 12 months) factors. We assumed an individual had been infected with a non-HIV STI infection if they reported being diagnosed with an STI and/or having a genital sore/ulcer and/or having a genital discharge. Many socioeconomic factors in the bivariate analysis contain information implicitly related to age, so we used an age-adjusted model to account for the peak in age-stratified HIV prevalence (that is, odds ratios were adjusted for age and age^2^). We calculated age-adjusted odds ratios (aORs) and 95% CIs. Separate analyses were conducted for men and women. For men, we also analyzed DHS data on the age of circumcision and the method that had been used (traditional or medical). Finally, we conducted a multivariate logistic regression analysis. In all analyses, covariates with *P* ≤0.05 were considered to be statistically significant.

## Results

**Figure 2:**
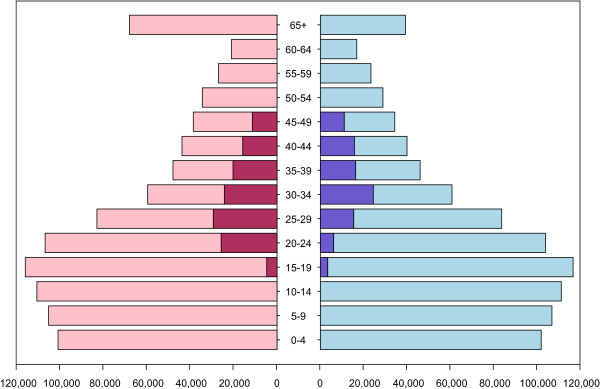
**Population distribution and HIV prevalence.** Population data are from the 2006 Census for women (pink) and men (light blue). HIV prevalence data, from the 2009–2010 Demographic and Health Survey is shown for women (red) and men (blue) aged 15 to 49 years old.

Approximately half of the population is younger than 20 years old, with approximately 40% younger than 15 years old; Figure 2 shows the HIV prevalence in five-year age classes for men and women. Forty-five percent of women are 15 to 49 years old and 49% of men are 15 to 59 years old. Prevalence is low in individuals younger than 20 (4% in women, 3% in men) but increases rapidly with age (Figure 2). In general, prevalence is greater in women than in men at all ages. The greatest gender difference (based on a five-year age stratification) is in individuals aged 20 to 24 years old, where prevalence is 24% in women and only 6% in men. Notably, prevalence peaks later in women than men: in 35 to 39 year-old women versus 30 to 34 year-old men.

**Figure 3:**
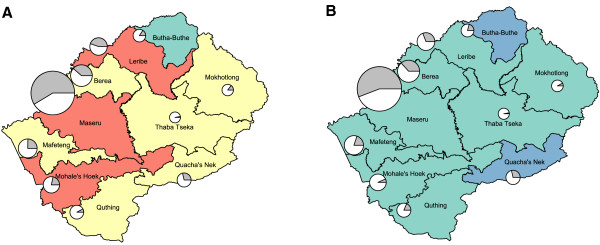
**Cartographic maps of HIV prevalence shown for healthcare districts and stratified by gender.** HIV prevalence is characterized for each healthcare district for **(A)** women and **(B)** men into the following categories: 11 to 16% (blue), 17 to 21% (green), 22 to 26% (yellow), 27 to 31% (red). For each healthcare district, the population in the urban center is indicated by the size of the circle. Within each circle, the gray region represents the proportion of HIV-positive individuals who live in the urban center, and the white represents the proportion that live in rural areas.

HIV prevalence is relatively uniform across the four ecological zones, ranging from 21% in the Senqu River Valley to 24% in the Lowlands. The gender-specific cartographic maps in Figure 3 show the geography of the epidemic in Lesotho in the 10 healthcare districts: prevalence in women is shown in Figure 3A, and prevalence in men is shown in Figure 3B. Notably, we found substantial geographic variation in prevalence among the 10 districts ranging from 16% in Butha-Buthe to 27% in Maseru. In each district prevalence is higher in women (range: 21% to 31%) than men (range: 11% to 20%). Prevalence is higher in urban centers (average 27%: women 31% and men 21%) than in rural areas (average 21%: women 24% and men 17%). The size of the circle in each district reflects the total number of individuals (HIV-infected and uninfected) living in the urban centers. With the exception of the Maseru district, which contains the capital city, few individuals live in urban areas (Figure 3). In each district, the size of the gray region within the circle reflects the proportion of HIV-infected individuals who live in the urban center. In the Maseru district, the majority (58%) of HIV-infected individuals live in the urban center. However, in the other nine districts the majority live in rural areas, ranging from 60% in Leribe to 98% in Thaba-Tseka.

Table 1 shows the demographic characteristics of men and women who were tested for HIV and completed the DHS individual survey. We note that these characteristics are shown for all adults whether they reported they were sexually active or not. HIV prevalence estimates were calculated using the sample weights provided by the DHS so that the survey cohort is representative of the national population. Overall prevalence is substantially higher in women than men (27% versus 18%). Nearly all women reported some education, with almost half receiving some degree of secondary education. Men were generally less educated than women. HIV prevalence was not associated with the level of education for either gender. Most women (55%) reported they were not employed over the past year, while most men (71%) reported some level of employment. Prevalence was significantly greater in both sexes who reported at least some employment over the past year in comparison with unemployed individuals. There was no significant difference in HIV prevalence between married women and women who had never been married (26% versus 24%). However, HIV prevalence in married men was substantially greater than in men who had never been married: 28% versus 8%. Not surprisingly, prevalence was significantly higher in widows (60%) and widowers (52%). The majority of respondents (95%) were either Catholic or Protestant. No significant difference was found in prevalence based on religion. Few individuals (15% of women, 13% of men) reported they had never had sexual intercourse. In these individuals prevalence was only 4% in women and 3% in men. Most respondents (86% of women, 87% of men) reported they had been sexually active in the past four weeks. Prevalence was significantly lower in the 5% of women who reported they were pregnant in comparison with women who reported they were not pregnant or were unaware they were pregnant: 18% versus 27%.

**Table 1:** Demographic characteristics by weighted HIV prevalence and gender, N = 6,924

	**Females**				**Males**			
	**N**	**#HIV+**	**%HIV+**	**95% CI**^**a**^	**N**	**#HIV+**	**%HIV+**	**95% CI**
**Total**	3,849	997	26.7	(25.3, 26.7)	3,075	543	18.4	(17.1, 19.8)
**Age (years)** median = 26	median = 26				median = 26			
IQR^b^ = (20, 36)	IQR = (20, 36)				IQR = (19, 37)			
**Age group (years)**								
15 to 19	895	63	4.2	(2.9, 5.5)	797	19	2.8	(1.7, 3.9)
20 to 29	1,314	379	28.9	(26.4, 31.4)	956	141	11.2	(9.2, 13.2)
30 to 39	842	359	41.4	(38.1, 44.7)	554	194	38.3	(34.3, 42.3)
40 to 49	601	191	32.8	(29.0, 36.6)	363	122	35.8	(30.9, 40.7)
50 to 59	—	—	—	—	265	63	23.1	(18.0, 28.2)
**Education**								
No education	55	15	20.5	(9.8, 31.2)	472	121	25.2	(21.3, 29.1)
Primary incomplete	1,083	294	28.7	(26.0, 31.4)	1,203	197	17.6	(15.4, 19.8)
Primary complete	901	266	31.7	(28.7, 34.7)	354	67	19.2	(15.1, 23.3)
Secondary+	1,810	422	23.8	(21.8, 25.8)	1,046	158	16.7	(14.4, 19.0)
**Employment status**								
No	2,124	376	21.1	(19.4, 22.8)	904	88	9.9	(8.0, 11.8)
In the past year	315	56	31.0	(25.9, 36.1)	202	39	21.3	(15.7, 26.9)
Currently working	1,339	236	33.8	(31.3, 36.3)	1,969	416	22.1	(20.3, 23.9)
Have a job (on leave)	70	12	31.7	(20.8, 42.6)	—	—	—	—
**Marital status**								
Never married	1,291	196	15.7	(13.7, 17.7)	1,595	115	7.5	(6.2, 8.8)
Married	2,057	506	25.8	(23.9, 27.7)	1,257	333	28.6	(26.1, 31.1)
Living together	30	23	75.0	(59.5, 90.5)	22	8	37.0	(16.8, 57.2)
Widow/Widower	299	171	59.4	(53.8, 65.0)	94	54	55.7	(45.7, 65.7)
Divorced/Separated	172	101	59.4	(52.1, 66.7)	107	33	29.7	(21.0, 38.4)
**Religion**								
Catholic	1,630	435	27.4	(25.2, 29.6)	1,268	230	18.6	(16.5, 20.7)
Protestant	2,117	536	26.4	(24.5, 28.3)	1,569	273	18.7	(16.8, 20.6)
Muslim/Other	68	19	24.6	(14.4, 34.8)	48	5	12.0	(2.8, 21.2)
None	34	7	23.1	(8.9, 37.3)	190	35	17.6	(12.2, 23.0)
**Sexual activity**								
Never had intercourse	551	23	3.2	(1.7, 4.7)	385	13	3.9	(2.0, 5.8)
Active (last 4 weeks)	1,444	402	30.4	(28.0, 32.8)	1,355	315	24.8	(22.5, 27.1)
Not active (last 4 weeks)	—	—	—	—	1,292	202	15.7	(13.7, 17.7)
- postpartum abstinence	420	90	20.2	(16.4, 24.0)	—	—	—	—
- not postpartum abstinence	1,394	465	33.3	(30.8, 35.8)	—	—	—	—
**Pregnancy**								
No or unsure	3,667	963	27.2	(25.8, 28.6)	—	—	—	—
Yes	182	34	17.7	(12.2, 23.2)	—	—	—	—

^a^95% CI, 95% confidence intervals.

^b^IQR, interquartile range.

Table 2 shows the age-adjusted factors associated with HIV infection by gender among the sexually active women (N = 3,243) and men (N = 2,546). Marital status, lifetime number of sex partners, condom use, having received an injection within the past year and having an STI within the past year were all (for both genders) significantly associated with HIV infection. For women, tobacco usage and pregnancy were also associated factors. For men, but not for women, having taken an HIV test was associated with HIV infection. Approximately 40% of women reported only one lifetime sex partner, approximately 50% reported two to four lifetime sex partners, and only 9% reported more than five. Notably, although prevalence increased with the number of partners, there is a very high prevalence (18%) in women with only one lifetime sex partner; it is also fairly high (12%) in men who reported only one (or one to two) lifetime sex partner(s). Thirty percent of men reported one to two lifetime sex partners, almost half reported three to six lifetime sex partners, and 25% reported more than seven. Condom use was fairly high: 34% of women and 46% of men reported condom use during their most recent sexual intercourse. A high proportion of women (31%) and a much lower proportion of men (13%) reported receiving an injection in the past year. The prevalence of an infection with a STI over the past year was similar in women (14%) and in men (13%).

**Table 2:** Age-adjusted risk factors associated with HIV infection by gender among sexually active participants, N = 5,789

		**Females**					**Males**				
		**N**	**% HIV+**	**aOR**^**a**^	**95% CI**^**b**^	***P-value***	**N**	**% HIV+**	**aOR**	**95% CI**	***P-value***
**Total**		3,243	30.6	—	—	—	2,546	19.7	—	—	—
**Age (years)** median = 28		median = 28					median = 28				
IQR^c^ = (21, 37)		IQR = (22, 37)					IQR = (21, 38)				
**Demographic characteristics**											
*Marital Status*											
Never married		732	24.4	ref	—	—	1,180	8.2	ref	—	—
Married		2,020	25.5	0.6	(0.5, 0.8)	<0.001	1,168	28.1	1.5	(1.1, 2.0)	0.009
Living together		29	77.8	7.1	(2.9, 19.9)	<0.001	21	37.0	2.3	(0.8, 6.1)	0.099
Widowed		292	60.2	2.7	(1.9, 3.7)	<0.001	81	52.0	5.2	(3.0, 9.0)	<0.001
Divorced/Separated		170	60.1	2.8	(1.9, 4.1)	<0.001	96	30.9	1.9	(1.1, 3.1)	0.017
*Mobility*											
Away for >1 month (last 12 mos.)		611	32.9	1.4	(1.1, 1.8)	0.003	581	19.4	0.8	(0.6, 1.1)	0.234
**Sexual behavior characteristics**											
Number of lifetime sexual partners for:											
Females:1	Males:1 to 2	1,259	17.5	ref	—	—	718	12.4	ref	—	—
2 to 4	3 to 6	1,637	36.0	2.4	(2.0, 2.9)	<0.001	1,051	17.6	1.4	(1.0, 1.9)	0.024
≥5	≥7	310	55.1	4.5	(3.4, 6.0)	<0.001	661	28.9	1.8	(1.4, 2.5)	0.001
Used condom at last intercourse		910	41.8	2.2	(1.8, 2.6)	<0.001	960	21.1	1.9	(1.5, 2.4)	<0.001
Ever paid for sex		—	—	—	—	—	146	29.4	1.4	(0.9, 2.1)	0.098
Paid for sex (last 12 mos.)		—	—	—	—	—	68	29.1	1.6	(0.9, 2.7)	0.124
Currently pregnant		179	17.9	0.7	(0.4, 1.0)	0.035	—	—	—	—	—
**Other risk characteristics**											
Ever tested for HIV		2,389	30.9	0.9	(0.8, 1.1)	0.299	1,093	26.7	1.5	(1.2, 1.9)	<0.001
Received injection (last 12 mos.)		932	33.6	1.3	(1.1, 1.5)	0.004	307	28.9	1.7	(1.2, 2.2)	0.001
Uses tobacco		442	43.6	1.4	(1.1, 1.7)	0.004	956	21.8	1.1	(0.9, 1.3)	0.580
Ever drank alcohol		—	—	—	—	—	1,467	22.3	1.2	(0.9, 1.5)	0.177
STI symptoms (last 12 mos.)		480	42.7	1.7	(1.4, 2.1)	<0.001	342	31.3	2.2	(1.6, 2.9)	<0.001
**Male Circumcision (MC) categories**											
Circumcised		—	—	—	—	—	1,558	20.3	0.9	(0.7, 1.1)	0.321
*Circumcision type:*											
Uncircumcised		—	—	—	—	—	988	18.9	ref	—	—
Traditional method		—	—	—	—	—	1,434	20.9	0.9	(0.7, 1.2)	0.485
Healthcare method		—	—	—	—	—	120	14.1	0.6	(0.3, 1.0)	0.053
*Sexual debut *and *MC:*											
Uncircumcised		—	—	—	—	—	988	18.9	ref	—	—
Sex before MC		—	—	—	—	—	556	19.5	0.8	(0.6, 1.0)	0.053
Same year		—	—	—	—	—	200	24.0	1.2	(0.8, 1.7)	0.478
MC before sex		—	—	—	—	—	802	20.0	1.0	(0.7, 1.2)	0.715

^a^aOR, age-adjusted odds ratio: ~ AGE + AGE^2^. Females 15 to 49 years old; males 15 to 59 years old.

^b^95% CI, 95% confidence intervals.

^c^IQR, interquartile range.

**Figure 4:**
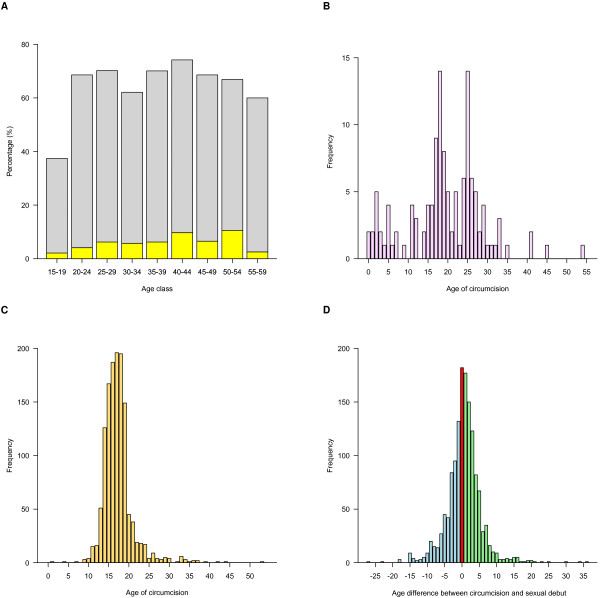
**Age distribution for factors characterizing male circumcision in Lesotho. (A)** Prevalence of medical (yellow) and traditional (gray) circumcision stratified by age. **(B)** Age at which medical circumcision occurred. **(C)** Age at which traditional circumcision occurred. **(D)** The number of years between the age at which traditional circumcision occurred and the age of sexual debut: circumcision before sexual debut (green), circumcision and sexual debut in the same year (red), circumcision after sexual debut (blue).

Table 2 shows the age-adjusted factors related to circumcision and their association with HIV infection. Notably, HIV prevalence in circumcised and uncircumcised men is approximately equal (approximately 20%). A high percentage (52%) of men reported they were circumcised, but only 9% reported the procedure had been performed by a healthcare professional (that is, medical circumcision). Prevalence of circumcision increases with age from 34% in men 15 to 19 years old to a high of 61% in men 20 to 59 years old (Figure 4A). The age at which medical circumcision occurred varies considerably (Figure 4B). However, the age of traditional circumcision, which is performed at a tribal initiation ceremony, is tightly distributed around 18 years old (IQR: 16 to 20) (Figure 4C). Almost half of the circumcised men had been circumcised before becoming sexually active; the median age of sexual debut for all men was 17 years old. The other half had been circumcised soon after their sexual debut (Figure 4D). We did not find statistical significance in the bivariate analysis on the age difference between sexual debut and circumcision (Table 2).

Table 3 shows the results of the multivariate logistic regression analysis. For both genders, three factors were found to be associated with an increased risk of HIV infection: the lifetime number of sex partners, infection with an STI in the past year, and receiving an injection in the past year. The number of lifetime sex partners is the most substantial risk factor. For women with 2 to 4 partners, the aOR is 2.3 (CI: 1.9-2.8) and for women with ≥5 partners, it is 4.4 (CI: 3.3-5.8). For men with 3 to 6 partners the aOR is 1.4 (CI: 1.0-1.9) and for men with ≥7 partners it is 1.8 (CI: 1.3-2.5). Respondents who reported an STI over the past year had an increased risk of HIV infection (women: aOR 1.6 (CI: 1.3-1.9); men: aOR 1.9 (CI: 1.3-2.5)). Individuals who reported receiving an injection in the past year were also more likely to be infected (women: aOR 1.3 (CI: 1.1-1.5); men: aOR 1.7 (CI: 1.2-2.3)). Women who reported they were pregnant were less likely to be infected (aOR 0.6 (CI: 0.4-1.0)) than women who were not pregnant or who were unaware they were pregnant. Medical circumcision was found to be protective (aOR 0.5 (CI: 0.3-0.9)), but traditional circumcision was not (aOR 0.9 (CI: 0.7-1.2)).

**Table 3:** Multivariate logistic regression risk factor analysis for HIV infection

		**Females**			**Males**		
		**aOR**^**a**^	**95% CI**^**b**^	***P-value***	**aOR**	**95% CI**	***P-value***
**Sexual behavior covariates**							
Females:1	Males:1 to 2	ref	—	—	ref	—	—
2 to 4	3 to 6	2.3	(1.9, 2.8)	<0.001	1.4	(1.0, 1.9)	0.027
≥5	≥7	4.4	(3.3, 5.8)	<0.001	1.8	(1.3, 2.5)	<0.001
**Other covariates**							
Currently pregnant		0.6	(0.4, 1.0)	0.046	—	—	—
Received injection (last 12 mos.)		1.3	(1.1, 1.5)	0.029	1.7	(1.2, 2.3)	0.002
Reported STI (last 12 mos.)		1.6	(1.3, 1.9)	0.001	1.9	(1.4, 2.6)	<0.001
*Circumcision type:*							
Uncircumcised		—	—	—	ref	—	—
Traditional method		—	—	—	0.9	(0.7, 1.2)	0.355
Healthcare method		—	—	—	0.5	(0.3, 0.9)	0.043

^a^aOR, age-adjusted odds ratio: ~ AGE + AGE^2^. Females 15 to 49 years old; males 15 to 59 years old.

^b^95% CI, 95% confidence intervals.

## Discussion

Our results show there are several important drivers of the Lesotho epidemic, with the most important risk factor being the number of lifetime sex partners. We found risk of infection with HIV increases substantially, for both women and men, as the number of lifetime partners increases. However, notably, our results show that - unlike in other HIV epidemics in Sub-Saharan Africa [16-18] - it does not appear to be necessary to have many sex partners to become infected with HIV. In Lesotho, nearly one in five women who report only one lifetime sex partner is infected. This is substantially higher than in other Sub-Saharan countries, such as Kenya, where the prevalence of HIV in women with only one lifetime partner is 9% [16]. The high risk of infection for women in Lesotho with only a few sex partners results from the hyperendemic prevalence level (that is, even with only one partner there is a high probability the partner is infected). The high prevalence of other STIs is also an important driver of the HIV epidemic in Lesotho, as in other countries in Sub-Saharan Africa [16-20].

Our results regarding the association of increased risk of HIV infection with injections and pregnancy should be interpreted with caution. As in any cross-sectional study we cannot identify causality, nor can we control for all possible confounders. Injections may have been unsafe and, consequently, a risk factor for HIV infection, or they may simply be an associated factor; for example, after becoming infected, individuals may have been more likely to receive injections for medical reasons than uninfected individuals. HIV-infected women are known to have a lower fertility rate than uninfected women; this may be the reason that we found pregnancy to be associated with a decreased risk of HIV infection.

In Lesotho, antiretroviral therapies for HIV have been available since 2001; however, current coverage is less than 60% among those with a CD4 count below the current treatment initiation threshold of 350 cells/μL [8,21]. By 2015, the Government aims to increase coverage to 90% of those in need, based on the current treatment threshold. They also aim to provide treatment to HIV-infected individuals with active TB regardless of their CD4 cell count. Notably, the World Health Organization (WHO) has recently recommended a new treatment initiation threshold of 500 cells/μL [22]. If this new threshold is used in Lesotho, the number of individuals in need of treatment will increase substantially. The cartographic country-level HIV prevalence maps we have constructed show the current geographic distribution of the HIV-infected population. They show that, although prevalence is higher in urban centers than rural areas, the overall burden of disease is greatest in rural areas. This is where increasing treatment coverage will be extremely challenging. The maps can be used at the country-level and/or the district level to estimate the total number of individuals who are currently (or soon will be) in need of treatment. Therefore, the maps can be used to identify where there is the greatest need for additional treatment programs, and to assess the optimal geographic location for new treatment clinics. As treatment coverage increases, incidence is likely to decrease (as treated individuals are less infectious than untreated individuals) [23,24]. However, since HIV in Lesotho is hyperendemic, the effect of treatment on reducing incidence is likely to be fairly gradual.

Lesotho’s current national guidelines for HIV/AIDS prevention include a comprehensive plan that aims to reduce the number of HIV transmissions by 50% by 2015 [9]. Planned behavioral interventions include: an increase in testing for HIV, a decrease in alcohol consumption and a reduction in sexual risk behaviors (that is, delaying the age of sexual debut, increasing condom use, decreasing the number of sex partners and reducing concurrent partnerships). Rolling out a combination package based on these interventions could potentially significantly reduce HIV transmission. However, we have found that a very large number of women in Lesotho (approximately 20%) have become infected with HIV but have had only one lifetime sex partner. This indicates that behavioral interventions that target women and aim to reduce their number of sex partners may not be as effective in reducing transmission in Lesotho as in other countries in Sub-Saharan Africa. Our results also imply that many women in Lesotho may acquire HIV infection from their husbands and are initially the uninfected partner in a discordant partnership. This is in accord with our previous results where we have shown that stable discordant couples may account for a substantial proportion of the new infections (that is, incidence) in Lesotho [25]. We have also shown that targeting stable discordant couples - unlike in many countries in Sub-Saharan Africa - would be feasible in Lesotho [26]. However, preventing infection in these couples will be challenging and a combination of behavioral and biomedical interventions will be necessary. We have shown that using treatment to prevent infection in stable discordant couples could significantly reduce transmission [27], but increasing condom usage, microbicides and pre-exposure prophylaxis should also be considered.

Interventions based on medical circumcision are now being rolled out in many countries in Sub-Saharan Africa. The World Health Organization has prioritized this intervention in Lesotho and scale-up has begun [9,28]; but to date only approximately 13,000 men have been circumcised. We have found that medical circumcision is very protective against HIV infection in Lesotho, but that the current prevalence of medical circumcision is less than 5%. These results indicate that circumcision has had very little effect on preventing the rise of the HIV epidemic. We have also found that traditional circumcision does not protect against HIV infection. This result is not surprising, because circumcision at an initiation ceremony generally involves only partial removal of the foreskin [29] and often occurs (as we have found) after men have become sexually active. Notably, we found that the current prevalence of traditional circumcision in Lesotho is very high (approximately 40%); consequently, reaching the 80% target for medical circumcision in adults, as recommended by UNAIDS and the WHO, will be extremely difficult [30]. Any medical circumcision plan for Lesotho must be comprehensive. It is important to try to design creative interventions based on “re-circumcision” of men who have been circumcised in tribal initiation ceremonies, as well as to target men younger than 25 years old. Attaining the target of 100% neonatal medical circumcisions in newborns is more feasible, but will depend on whether parents are willing to change cultural practices.

## Conclusions

The epidemic in Lesotho is one of the most severe HIV epidemics worldwide, but surprisingly has received little attention. Our results have shown that there is a need for a substantial expansion of testing and treatment programs throughout the country, particularly in rural areas. This will be extremely difficult due to the mountainous terrain, the poor transportation network, the limited health care infrastructure and scarce financial resources. Testing will need to be frequent and expansive. Notably, over a third of the population in Lesotho is under 15 years old and many individuals will soon become sexually active. There is a critical need for the implementation of effective intervention programs for reducing HIV transmission. Our analyses provide key scientific insights into understanding this epidemic, and can be used as the basis for choosing among intervention strategies. Our results suggest that some public health interventions that may be very effective in reducing transmission in other Sub-Saharan African countries (particularly interventions aimed at reducing the number of sex partners, or based on medical circumcision) may not be very effective in Lesotho. More expensive biomedical interventions based on microbicides, pre-exposure prophylaxis and “treatment as prevention” may need to be considered. Clearly, mitigation efforts based on a combination of interventions will be necessary. HIV in Lesotho is hyperendemic and will be very difficult to control; however, it is essential to intervene, and to intervene quickly.

## Abbreviations

aORs: Age-adjusted odds ratios; ARVs: Antiretrovirals; CIs: Confidence intervals; DHS: Demographic and health survey; IQR: Interquartile range; MC: Male circumcision; STIs: Sexually transmitted infections; WHO: World health organization.

## Competing interests

The authors declare that they have no competing interests.

## Authors’ contributions

BJC, JTO and SB developed the concept and study design, analyzed and interpreted the data and drafted the manuscript. BJC and JTO conducted the statistical analyses. SB supervised the project. All authors read and approved the final manuscript.

## Pre-publication history

The pre-publication history for this paper can be accessed here:

http://www.biomedcentral.com/1741-7015/11/224/prepub
